# Interprofessional care of patients with type 2 diabetes mellitus in primary care: family physicians’ perspectives

**DOI:** 10.1186/s12875-022-01688-w

**Published:** 2022-04-08

**Authors:** Jacqueline M. I. Torti, Olga Szafran, Sandra L. Kennett, Neil R. Bell

**Affiliations:** 1 Department of Medicine, Schulich School of Medicine and Dentistry; Faculty of Education; Centre for Education Research and Innovation, Schulich School of Medicine and Dentistry, Western University. Medical Sciences Building Suite 102A, London, ON N6A 5C1 Canada; 2 Department of Family Medicine, University of Alberta, 6-10 University Terrace, Edmonton, AB T6G 2T4 Canada; 3 Clinical Nurse Specialist, Edmonton Oliver Primary Care Network Family Medicine Clinic, Presently Is: Nurse Advisor, Primary Care, Health Canada, Misericordia Community Hospital, Edmonton, Alberta, Canada, Suite 730, 9700 Jasper Avenue, Edmonton, AB T5J 4C3 Canada; 4 Department of Family Medicine, University of Alberta Family Medicine Clinic, Misericordia Community Hospital, 16940 - 87 Avenue, Edmonton, AB T5R 4H5 Canada

**Keywords:** Patient care team, Family physician, Primary health care, Qualitative research, Type 2 diabetes mellitus, Medication therapy management

## Abstract

**Background:**

There is a lack of understanding of the team processes and factors that influence teamwork and medication management practices in the care of patients with type 2 diabetes mellitus (T2DM). The purpose of the study was to explore physicians’ perspectives of barriers and facilitators to interprofessional care of patients with T2DM within team-based family practice settings.

**Methods:**

This was a qualitative, descriptive study. Participants included physicians affiliated with a primary care network providing care to patients with T2DM in an interprofessional team-based primary care setting in Edmonton, Alberta, Canada. Participants’ contact information was obtained from the publicly available College of Physicians and Surgeons of Alberta and respective primary care network websites. Interview questions addressed physicians’ perspectives on factors or processes that facilitated and hindered the care and medication management of adult patients with T2DM in primary care team-based clinical practice. Interviews were audio-recorded, transcribed, and analyzed using qualitative content analysis and a constant comparative approach.

**Results:**

A total of 15 family physicians participated in individual interviews. Family physicians identified facilitators of interprofessional team-based care and medication management of patients with T2DM in three theme areas—access to team members and programs, knowledgeable and skilled health professionals, and provision of patient education by other health professionals. Two themes emerged as barriers to interprofessional care – lack of provider continuity and the loss of skills from delegation of tasks.

**Conclusion:**

Family physicians perceive both benefits and risks to interprofessional team-based care in caring for patients with T2DM. Successful functioning of team-based care in family practice will require overcoming traditional professional roles.

**Supplementary Information:**

The online version contains supplementary material available at 10.1186/s12875-022-01688-w.

## Background

There are approximately 3 million Canadians living with diabetes, with estimates as high as 11 million when undiagnosed and pre-diabetes are considered. [1, 2] Diabetes and pre-diabetes are lifelong chronic conditions that increase the risks of related health complications. [3] Diabetes prevalence increases with age and, with an ageing population, this will place a significant burden on Canada’s health care system and economy. [1] Inter professional team-based diabetes management aims to improve patient outcomes and use scarce health care resources efficiently. [4] The care of patients with type 2 diabetes mellitus (T2DM) can be complex and challenging for patients, families and the health care system. [3, 5] In order to optimize care of patients with T2DM, the World Health Organization, governments, health care organizations, and health care experts, advocate for a collaborative, multifaceted, and multiple strategy approach. [6].

A key feature of primary care reform in Canada is the integration of team-based care that includes various health professionals in the provision of comprehensive services to patients. Family physicians have traditionally been the main primary care providers. They are now integrating various health professionals into their practices to deliver care in an inter professional team-based model of care. [7] This model of care has family physicians working alongside other primary care providers such as nurses, pharmacists, social workers, and community organizations to provide primary care services that enhance patient care through a team-based approach. [7] The transition to primary health care teams, however, does come with challenges. Inter professional teams have encountered difficulties in understanding the roles and responsibilities of various health professionals, particularly in the early stages of implementation. [8] Inter professional teams are expected to collaborate, coordinate, and make shared decisions when caring for patients. While team outcomes are important, team structure, team relationships, team processes and organizational factors may be more robust indicators of positive outcomes for team effectiveness in the primary care setting. [9, 10] Inter professional team processes include both interpersonal and professional issues. Team processes can consist of coordination, communication, conflict resolution, decision-making, problem-solving, and boundary sparring. [11].

Although there have been challenges to implementing inter professional teams within primary care settings, the team approach has shown to have a positive effect on patient outcomes with diabetes. [12–15] In a recent study that occurred one year after the initiation of inter professional teams in Alberta, patients were less likely to be admitted to a hospital or to visit an emergency department for diabetes-specific care than those whose care was not managed by a team of health professionals. [14] In addition, patients with T2DM who were cared for by an inter professional team were more likely to see an ophthalmologist or optometrist and to undergo guideline-recommended laboratory investigations. [13, 14] Improvements in patient outcomes with diabetes appear to be driven by the coordination and collaboration of health professionals focused on delivering quality patient care based on the best available evidence. [13, 16].

Medication management of patients with T2DM includes components of medication reviews, reconciliation, preparation, administration, monitoring, adjustment, and education on the safe and efficacious use of medications. [17, 18] Studies indicate that a collaborative pharmacist and physician approach to medication management within primary care settings can improve processes of care and patient outcomes with diabetes. [17, 19–21] However, few studies have explored inter professional team-based care practices, processes, and facilitators and barriers to medication management in the treatment of patients with T2DM from the perspective of family physicians. [22–26] Although insights have been gained into the challenges of implementing and developing inter professional teams within the primary care setting, there is a lack of understanding of the team processes and factors that influence teamwork and medication management practices in the care of patients with T2DM. As such, the purpose of this study was to explore physicians’ perspectives of barriers and facilitators to inter professional team-based care of patients with T2DM within family practice settings.

## Methods

### Study design

A qualitative, exploratory, descriptive study approach employing individual interviews was used to explore family physicians’ perspectives on inter professional team-based care of patients with T2DM within the primary care setting. A qualitative descriptive approach was deemed most appropriate to explore and capture family physicians’ perspectives and subjective experiences because a clear description and basic level understanding of the phenomenon was desired. [27].

### Setting

This study was conducted with family practices affiliated with a Primary Care Network (PCN) in Edmonton, Alberta, Canada. At the time of the study, 42 PCNs were operating throughout Alberta (4 PCNs in Edmonton), with more than 3800 family physicians working in PCNs. PCNs were comprised of physicians, nurses, dietitians, pharmacists, and other allied health professionals working together as inter professional primary health care teams to provide care to patients. All PCNs shared common objectives, including improved access to primary care services, health promotion and disease prevention, and care of patients with chronic and complex health care needs. PCN services were offered through centralized and decentralized models. Most PCNs had identified chronic disease management as a priority and offered programs for the care of patients with T2DM, such as strategies for controlling blood sugar, healthy eating, meal planning, physical activity, foot care, insulin basics, and long-term management of T2DM. [12].

### Participant recruitment

Eligible participants included practicing family physicians who were recruited from family practices that were associated with PCNs. The sampling frame was comprised of 113 eligible family physicians—30 from four university academic teaching centres and 83 from community-based family practices. Physicians’ contact information was obtained from the College of Physicians and Surgeons of Alberta and the respective PCN websites. Physicians were faxed a recruitment notice to their place of business, inviting them to participate in an individual interview—those who expressed an interest in participating completed and returned a reply card. Non-respondents were followed up with a telephone call after one to two weeks to ensure they received the fax and inquire if they had any questions about the study. Physicians who returned a completed reply card were sent a study information letter and contacted to schedule an interview.

### Data collection

Individual interviews were conducted either in-person or via telephone by one of the research team members [JT], a trained qualitative researcher. The interviewer did not have a prior relationship with any study participants. A semi-structured interview guide (see Additional file 1) consisting of open-ended questions was used to enable physicians to express their thoughts, perceptions, and experiences with team-based care of patients with T2DM. Each interview consisted of questions regarding clinical practice, team-based care, and medication management for patients with T2DM. Participants were asked what factors or processes facilitated the care and medication management of adult patients with T2DM and what factors or processes hindered the care and medication management of adult patients with T2DM in their clinical practice. Interviews were scheduled at the convenience of the participants, were audio-recorded, and were transcribed by a professional transcription agency. The interviews were conducted in November of 2016 and ranged from 16 to 38 min in duration.

### Data analysis

Data analysis was performed using qualitative content analysis [28] and a constant comparative approach [29] with a social constructivist lens by two researchers [JT & OS] who independently read, reviewed and coded each transcript prior to meeting regularly to collectively analyze the qualitative data. Codes, categories, and subcategories were used to organize the analysis of the transcripts. The analysis involved in-depth group discussion in reaching a consensus on each code. After the two researchers had reached a consensus, the other two authors [SK & NB] were brought in to peer-review the preliminary analysis and provide feedback. JT and OS analyzed the data from a non-clinician perspective, while SK and NB offered different interpretations based on their role as health care providers. This analyst triangulation and expert checking contributed to improving the reliability of the study findings. [30].

## Results

A total of 15 family physicians took part in the study (8 males and 7 females) ranging in age from 33 to 62 years, with a mean age of 43 years. On average, participating physicians had 11 years of experience in clinical practice (range 1–34 years) working as a primary care provider. Nine of the physicians came from academic family medicine sites and the remaining 6 were from community-based family practices. All of the physicians had experience managing patients with T2DM within a primary care team-based setting.

## Facilitators

Participants identified facilitators in three theme areas for medication management and the care of patients with T2DM—accessibility to team members and programs, having knowledgeable and skilled team members, and supports available for patient education.

**Accessibility to Team Members & Programs.** Ease of access to team members and programs was noted to facilitate interprofessional care of patients with T2DM. Co-location, as facilitated by a decentralized PCN model, enabled physicians to share physical space with other health professionals, which helped to forge relationships and allowed for face-to-face communication. When team members were on-site and readily available, patient appointments were better coordinated with various PCN health professionals.

“…access to all of the support of the team members, the dietitian, the chronic disease management nurse, and pharmacists… everyone being in fairly close proximity.” Interview 10

“I think just having the multidisciplinarity in one setting, under one roof works very well, and to be able to refer to these people very quickly and easily is another thing that helps to facilitate patient management.” Interview 11

Physicians also found that when programs offered through the PCN were readily accessible within their clinic, it eased the process of coordinating patient referrals, eliminated long wait times, and expedited prompt care. Identifying the PCN programs that would best suit patient needs was further enabled by team members’ familiarity with the various programs.

“… it’s just immediate access to some of the services that are provided within the PCN, so you have more immediate access to other medical disciplines or professional disciplines within that team.” Interview 2

**Knowledgeable & Skilled Team Members.** Physicians expressed that having team members who are knowledgeable and competent in providing care and managing medications for patients with T2DM facilitated teamwork through the development of trust. Gaining an understanding of each team member’s knowledge and skills enabled physicians to develop confidence and comfort in entrusting the care of patients to these professionals. Part of effective teamwork is knowing who one’s team members are, their roles and responsibilities, and the skillset they bring to the team. A key understanding of these features enabled physicians to work effectively with team members and ensured team functioning was optimized for patient care.

“… our chronic disease nurse is very good at managing insulin, maybe is more understanding and knowledgeable about it than myself at times, and so it’s just really an excellent resource in terms of getting an opinion on what you should do with your patient regarding their diabetes and how we should manage it.” Interview 8

“I’d say by far her expertise in insulin management far outstrips my own knowledge and expertise. So, I would trust her assessments over that inclusively. Having a skilled accessible person who I can communicate with regularly, who I can do opportunistic care with our patients…” Interview 13

**Support for Patient Education.** Physicians articulated that they are often pressed for time or do not have the skillset that is necessary to educate patients with T2DM about illness management. The ability to share these responsibilities with other team members, such as the chronic disease management nurse and pharmacist, who possess the expertise and skillset necessary to meet a patient’s educational needs, significantly facilitated the care of patients with T2DM.

“So, within the team, the nurses and pharmacists I find are critical. For patients to truly understand it, because I find the physicians themselves don’t have enough knowledge regarding the devices, the monitoring devices, how to store, and manage them... I find the pharmacists and nurses do much better education regarding that.” Interview 2

“... that’s just value added to have the chronic disease management nurse be able to do that with the teaching that goes along with it and everything, which in the past personally I’ve never done, you’d always usually refer those out to the regional diabetes clinics, so it was lovely to keep that all within our medical clinic.” Interview 6

## Barriers

Barriers to teamwork in the care of patients with T2DM were noted in two theme areas, a lack of continuity of care providers and the loss of skills due to the delegation of tasks.

**Lack of Provider Continuity.** Having many providers involved in the care of a patient with T2DM was perceived to be a barrier to patient care by jeopardizing continuity of care. It was noted that when multiple health professionals provide inconsistent or conflicting care advice, patients become confused and lose trust. There is the possibility of miscommunication, disagreement, and discordance among providers, which can further complicate team functioning and patient care. This may adversely influence patient satisfaction and quality of care.

“Sometimes there’s too many cooks in the kitchen. And so, although the information that’s being provided by each of the disciplines is correct, it’s not... It can be overwhelming and incorrect information for the current problem. And so, trying to keep it specific to the current problem cause trouble when you have multiple disciplines.” Interview 2

“…a different pharmacist every day, or not a regular pharmacist, or not your usual regular permanent occupational therapy or physical therapy staff. It’s just hard to share information… they’re not as engaged [in] thinking what’s happened up till now and what the future plans are, and it becomes more fragmented…” Interview 1

**Loss of Skills from Delegation of Tasks.** Although physicians stressed the benefits of having reliable and skilled team members and support for patient education, they also commented on the potential downside of these aspects of team-based care. Physicians expressed that a reliance on other members of the primary care team meant that they did not spend as much time on certain aspects of T2DM care, such as patient education and medication management, and as a result, may lose some of the knowledge and skills that go along with those aspects of care. They largely rely on the chronic disease management nurse for ensuring evidence-based practice and being up to date on current best practices. The loss of skills was perceived to place physicians at a disadvantage in terms of supporting other team members.

“… as people become more dependent and more reliant on various team members to look after medication management, physicians need to be slightly aware of their potential to actually lose some of their skill set themselves in dictating and directing of medication management. So, I know it’s not uncommon to see our residents … coming out at the end of their block-time rotations saying, you know, I don’t actually really know how I should be starting insulin for patients … at the very least they need to have that core knowledge to be able to help support their team members when the need arises.” Interview 12

## Discussion

The study findings provide notable insights into family physicians’ perspectives on inter professional care and medication management of patients with T2DM in the primary care setting. Family physicians value the accessibility to knowledgeable and skilled health care professionals that is facilitated by co-location in a team-based model. Co-location is deemed to foster teamwork through the development of confidence and trust in the skillsets of other health professionals. This finding is consistent with other studies which found that decentralized models of care helped to foster team interaction and presented opportunities to discuss patient care, which ultimately led to trusting professional relationships. [31–33] Family physicians also value and appreciate the support provided by other health professionals for patient education on medication and illness management of T2DM. They acknowledge that they themselves do not possess the advanced skillset nor have the time for comprehensive patient education. This is supported by other studies that have reported on the delegation of tasks as a positive aspect of teamwork, including outcomes such as better patient education and freeing up physicians’ time. [22, 26, 31].

A new finding of our study is that family physicians feel that delegation of tasks to other members of the primary health care team can have negative consequences for themselves, including an over-reliance on others to provide care and the potential loss of their own skills and knowledge of best practices. While the concern over loss of knowledge and skills related to medication management and clinical care of patients is real, it may also reflect a reluctance to lose control or share care responsibilities with allied health professionals in the primary care setting. Traditionally, physicians have assumed greater legal responsibility for patient care and, thus, perceive themselves to have the leadership role in team-based care. [31, 34] Overcoming traditionally-engrained professional roles does pose a challenge to inter professional teamwork, including the inability of other health professionals to work to their full scope of practice. These challenges may be mitigated by developing an integrated inter professional model of teamwork that is based on trust, effective communication, clearly defined professional roles, and continuing inter professional development. [26, 35–37] If successfully implemented, inter professional teamwork can improve the quality of patient care, as well as increase patient and provider satisfaction.

The study findings have implications for practice and medical education. Given that family physicians value an inter professional team-based care approach to caring for patients with T2DM, it is important that identified barriers are mitigated through systems of support. Co-location should be a key consideration in the system design of inter professional clinical care teams. Joint professional development in the areas of medication management, insulin starts, foot care and dietary management, which includes physicians as well as allied health professionals, may alleviate concerns over loss of skills. Physicians’ concerns regarding loss of provider continuity may be addressed through frequent updates and reciprocal communication between all team members regarding the various aspects of patient care and patient status. This can include the use of shared electronic medical records and regular team rounding, common practices in primary care teams in Alberta. [31] In terms of medical education, this research highlights the importance of offering inter professional education opportunities to learners that can facilitate collaboration and an understanding of inter professional roles. The education system should also ensure that learners across all levels and disciplines have opportunities to train and work collaboratively in medication management of patients with T2DM.

The sample size of 15 participants in our qualitative study was deemed to be sufficient as data saturation appeared to have been reached with no new themes emerging. In addition, the analyst triangulation and expert checking that was integrated as part of the qualitative data analysis enabled multiple interpretations of the data and restrained selective perception. The study is limited, however, in that the findings are based solely on perceptions and not on observations of team-based interactions. Therefore, family physicians’ responses may be based more on personal reflections of facilitators and barriers rather than on enacted facilitators and barriers. The PCN model of team-based care employed in our study setting may not be reflective of other team-based primary care settings, as such, our findings may differ from other studies. It is likely that the study findings are relevant to the management of medical conditions other than T2DM in a team-based primary care setting, however, this is not certain. Our study reports on the perceptions of family physicians only and did not investigate the perceptions of other health care providers and patients. Future research would benefit from a more in-depth exploration of interprofessional teamwork from different members of the primary care team, in addition to in-clinic observations to supplement interview data.

Another consideration when interpreting the findings of our study should be that data were collected prior to the COVID-19 pandemic. With the onset of the pandemic, many physicians switched from office-based practice to telemedicine in a very short period. Although the balance between office-based practice and telemedicine is yet to be determined, it is very likely that a portion of visits by telemedicine will become a permanent part of primary care practices. This will need to be considered in managing and integrating team-based care for patients with T2DM. For example, with the uptake of virtual team meetings, there is less direct face-to-face interaction between team members, posing a major challenge to co-location. Virtual patient care may also make it more difficult to provide education to patients with T2DM because of constraints on their availability to use or demonstrate educational tools and materials. Telemedicine and the implementation of other technological solutions have also been reported to suffer from communication problems and can disrupt role clarity within interprofessional healthcare teams; these issues are further compounded by the complex nature of diabetes. [38] However, virtual care may also offer advantages to the care of patients with T2DM, including increased ability to set up initial and follow-up visits for patients that may find it difficult to attend in-person clinics. Future research would benefit from exploring the impact of virtual care on interprofessional care for patients with T2DM.

In addition to the shift to telemedicine, studies have also reported changes to inter professional team-based care in primary care practices in Canada as a result of the pandemic. For example, with the onset of the COVID-19 pandemic, primary care teams have experienced a rapid shift in team members’ roles which has implications for our study’s findings. [39] In addition, much of the support for chronic health conditions, such as diabetes, were not prioritized during the pandemic in primary care. [39] As a result, inter professional teams will likely be dealing with increased complexity in patient cases as they return to more routine care, further complicating the nature of these relationships. Yet, the pandemic has also increased inter professional care and, as a result, has highlighted the value of team-based models in primary care and team members' abilities to adapt to meet the needs of their patients. [39].

## Conclusion

Family physicians perceive both benefits and risks to interprofessional team-based care in caring for patients with T2DM. The study findings indicate that the successful functioning of team-based care in family practice will require health care providers to overcome traditional professional roles.

## Supplementary Information

**Additional file1:** 

## Data Availability

The datasets generated and/or analyzed during the current study are not publicly available due to the nature of a confidentiality agreement with study participants that only members of the study team will have access to the study data. However, for research purposes, de-identified study data are available from the corresponding author on reasonable request.

## Abbreviations

PCN: Primary Care Network
T2DM: Type 2 Diabetes Mellitus
