# Supplementary material for: Interprofessional care of patients with type 2 diabetes mellitus in primary care: family physicians’ perspectives
Source: BMC Prim Care. 2022 Apr 8;23:74. doi: 10.1186/s12875-022-01688-w (PMC8990268; doi:10.1186/s12875-022-01688-w)
Supplement: Supplementary file 1 — Additional file1: [file 12875_2022_1688_MOESM1_ESM.docx]

**Multidisciplinary team-based care and medication management in the treatment of patients with type 2 diabetes within primary care settings: Family physicians’ perspective**

**Interview Questions**

1. **Demographic Information**

- Male/female (Please circle one)
- Year of birth ________________
- Years of practice_________________

1. **Clinical Practice**

- Please describe the characteristics of your clinical practice. E.g. solo practice, team members, full-time/part-time, rural/urban.

1. **Group-Based Practice**

- What factors or characteristics facilitate multidisciplinary team-based care in your clinical practice?
- What factors or characteristics hinder multidisciplinary team-based care in your clinical practice?

1. **Type 2 Diabetes Care**

- What factors or processes facilitate the care of adult patients with type 2 diabetes in your clinical practice?
- What factors or processes hinder the care of adult patients with type 2 diabetes in your clinical practice?

1. **Medication Management with Type Diabetes Care**

- What factors or processes facilitate medication management with adult patients with type 2 diabetes in your clinical practice?
- What factors or processes hinder medication management with adult patients with type 2 diabetes in your clinical practice?

1. **Any additional comments you would like to share?**

Thank you very much for your time and participation.
